# Dr. Theron James

**Published:** 1913-06

**Authors:** 


Dr. Theron James, Buffalo, 1889, a practitioner of Medina, died at the Rochester General Hospital, May 4, after an illness of several months, aged 52. Before studying medicine he was a druggist in Medina. He was active in masonry and was a U. S. Pension Examiner from 1895 till his death.

